# Diagnosis Dilemma of Fibroblastic Osteosarcoma: A Case Report

**DOI:** 10.7759/cureus.74093

**Published:** 2024-11-20

**Authors:** Oraib Al-Rabadi, Hamzah H Alkofahi, Ribal Hamad, Tareg Al-Shishani, Muthana Al-Nawafla

**Affiliations:** 1 Oral and Maxillofacial Surgery, Jordanian Royal Medical Services, Amman, JOR; 2 Histopathology, Jordanian Royal Medical Services, Amman, JOR

**Keywords:** fibroblastic, jaws, mandible swelling, nodular fascitis, osteosarcoma

## Abstract

Osteosarcoma (OS) is a rare form of malignant bone tumor affecting jaws. The diagnosis of jaws osteosarcoma (JO) presents a unique challenge due to its rarity and the diversity of histological presentations it can exhibit. Fibroblastic osteosarcoma (FO), a subtype of OS, is characterized by the presence of fibroblastic cells and osteoid-producing cells within the tumor matrix. In this case report, we present a notable case of FO in a 27-year-old male patient who presented with a progressively enlarging swelling in the mandible. The lesion exhibited a diverse histological spectrum, displaying features reminiscent of benign proliferative lesions, which posed a diagnostic conundrum. However, the clinical presentation and radiological findings warranted further investigation. Subsequent biopsies were performed, revealing focal areas of osteoid production amidst the fibroblastic stroma, prompting reconsideration of the diagnosis. The presence of osteoid material, albeit minimal, raised suspicion for a malignant process, leading to a revised diagnosis of FO. The diagnostic challenge of FO underscores the importance of thorough sampling and histopathological examination in challenging cases, particularly in distinguishing between benign and malignant lesions in the jawbones.

## Introduction

Osteosarcoma (OS) is defined as a heterogeneous bone neoplasm exhibiting histopathologic features of malignant osteoid formation by atypical mesenchymal cells. The diagnosis confirmation hinges on the identification of direct osteoid formation, although minimal, by neoplastic osteoblasts. OS has a rare occurrence within the head and neck region, with the jawbones being the most frequently affected site [1]. Of these small percentage of cases, jaws osteosarcoma (JO) primarily occurs in the posterior region of the mandible [2,3]. Its peak incidence typically falls within the third to fourth decades, approximately a decade later than the peak incidence observed in long-bone OS [4]. Microscopically, about 50% of JO exhibit a chondroblastic subtype, characterized by minimal production of osteoid matrix or osteoblastic features. Furthermore, myxoid and fibroblastic variants represent lesser common histological subtypes of OS [5-9].

Clinically, swelling is the predominant presenting symptom in cases of OS, while other lesser frequent symptoms include pain, paresthesia, and ulcerations [10].

In this report, we describe a rare case of fibroblastic osteosarcoma (FO) in a 27-year-old male patient presenting with a mandibular swelling. The lesion exhibited diverse histologic features resembling proliferative reactive lesions. These findings posed a challenge for accurate pathologic diagnosis and effective surgical management.

## Case presentation

A 27-year-old male with no medical history presented with a gradually enlarging, painless, hard, and immobile mass on the left side of his face over the area of the mandibular angle for the past two months.

Extraoral examination showed facial asymmetry, hard swelling measuring 4 x 4 cm in size, and negative neck for cervical lymphadenopathy. Intraoral examination was negative for bony expansion or soft tissue mass on the left side of the mandible. Some of the patient's teeth on the same side exhibited simple caries, but none showed mobility. Also, the mucosa and gingiva on the left side of the mandible appeared normal.

**Figure 1:**
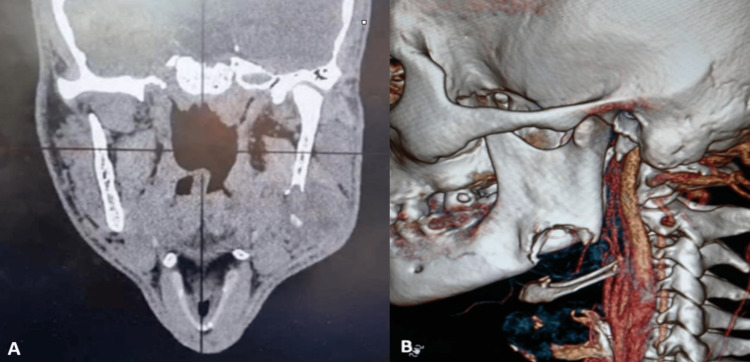
(A) A coronal computed tomography (CT) revealed a prominent 4 x 3 cm mass in the left mandibular angle area and cortical destruction was evident medially, laterally, and inferiorly, with extension into the surrounding soft tissues, (B) 3-D revealed a cortical destruction in the left mandibular angle.

Radiographically, the orthopantomogram (OPG) revealed a well-defined large lytic lesion located at the mandibular angle region, with noticeable destruction of the inferior cortical margin. No periapical pathology was observed in relation to the patient's teeth on the left side of the mandible. Further evaluation with computed tomography (CT) revealed a prominent 4 x 3 cm mass in the left mandibular angle area. Cortical destruction was evident medially, laterally, and inferiorly, with extension into the surrounding soft tissues (Figure 1).

In pursuing the confirmatory diagnosis, an incisional biopsy was performed under general anesthesia using an extraoral approach. Intraoperative findings revealed that the tumor originated from the mandibular bone, invading the surrounding muscles laterally and medially. Also, the mass was tethered with the masseter and medial pterygoid muscles. A specimen was obtained from the peripheral part of the tumor, fixed in formalin, and sent for histopathological examination.

**Figure 2:**
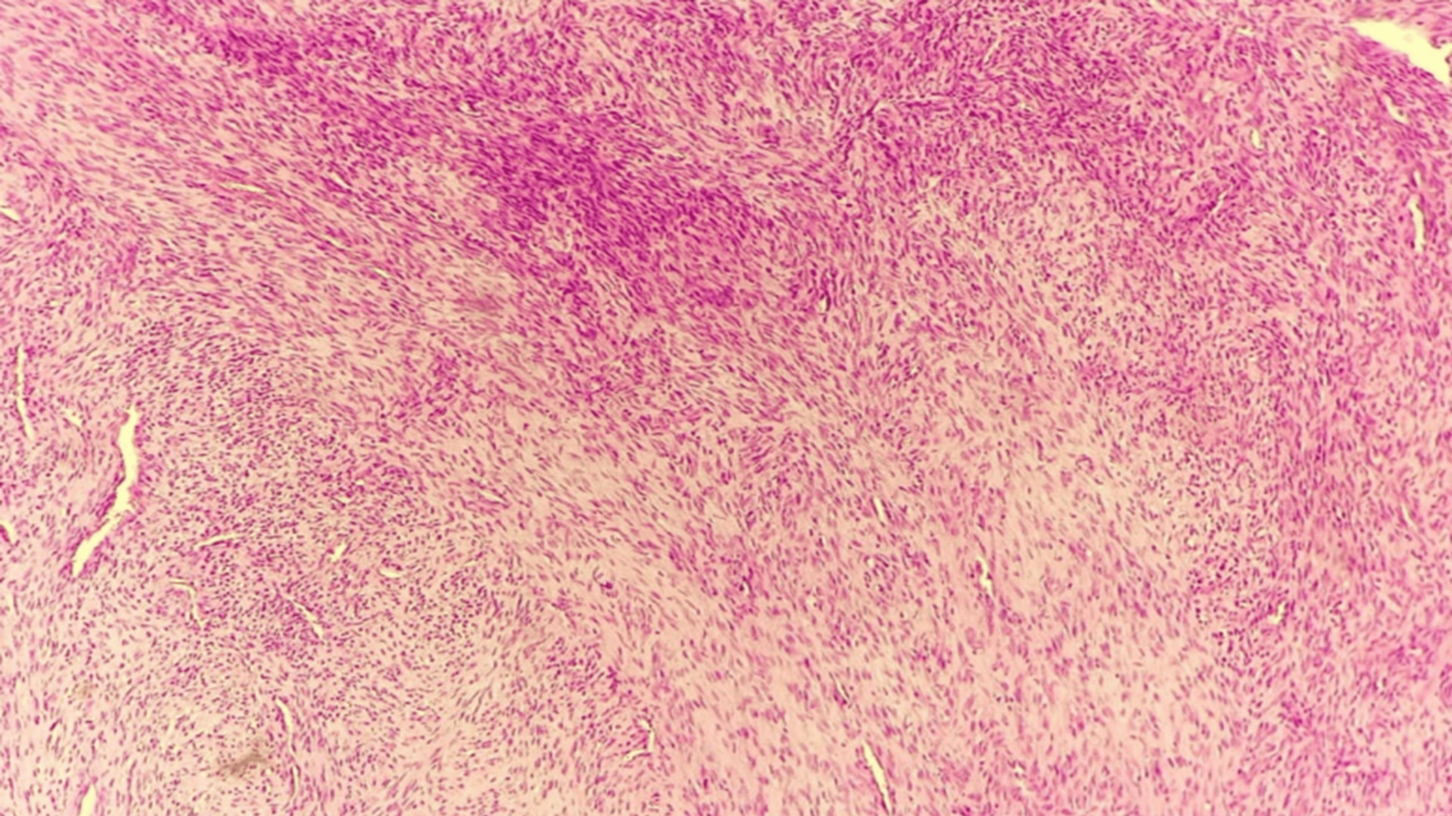
The section reveals bland spindled fibroblastic cells to stellate cells with a variably cellular tissue culture-like pattern, accompanied by extravasated red blood cells and pale nuclei with prominent nucleoli.

Microscopic analysis revealed bland spindled fibroblastic cells to stellate cells with a variably cellular tissue culture-like pattern, accompanied by extravasated red blood cells and pale nuclei with prominent nucleoli. Initially, the diagnosis was rendered as nodular fasciitis (Figure 2). However, given the suspicion raised by the correlation between CT findings and clinical characteristics of the tumor, a discussion ensued with the histopathologist. The consensus decision was to perform additional incisional biopsies from different parts of the tumor, particularly from its deeper regions. Subsequent biopsies were performed under general anesthesia, from deeper regions of the tumor. Microscopic examination revealed minimal osteoid production, prompting a change in diagnosis to FO.

**Figure 3:**
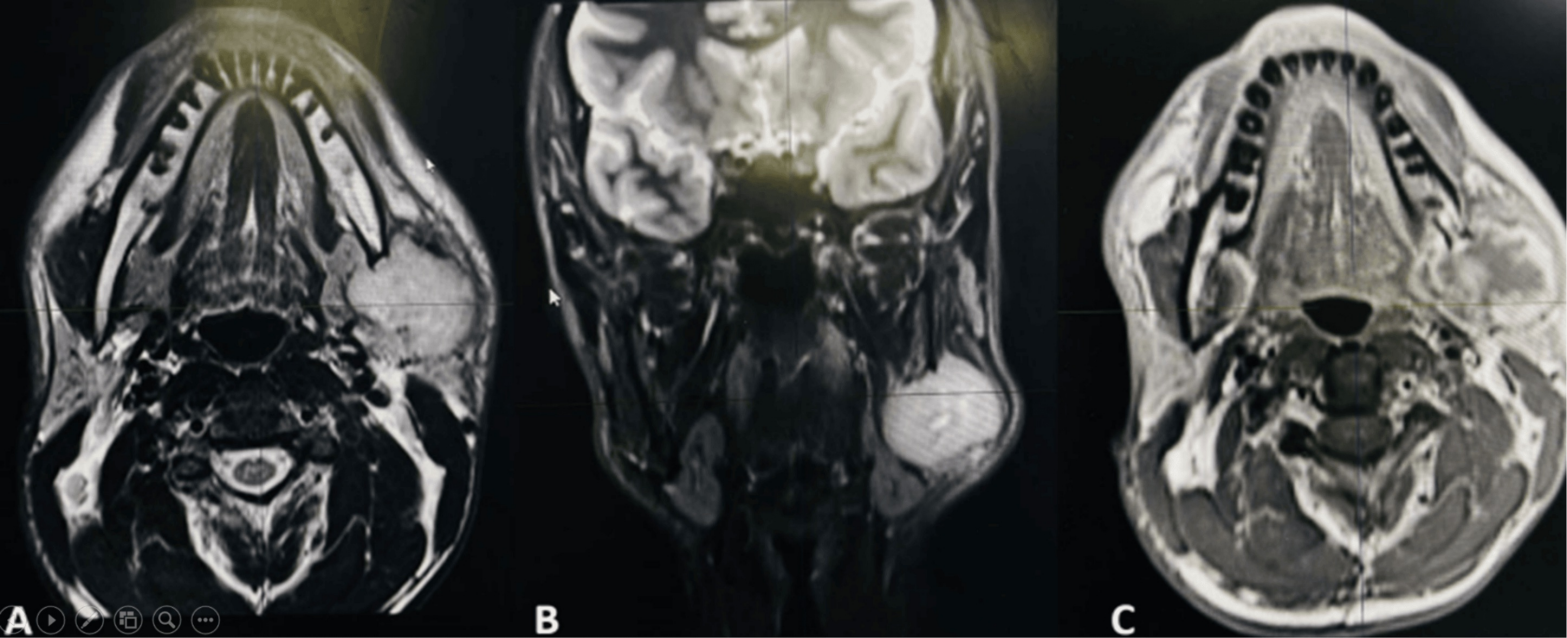
(A, B) T2 axial and coronal MRI revealed a 3.7 x 3.8 cm mass lesion originating from the angle of the mandible on the left side, associated with a soft tissue component involving both sides of the bone. The mass was observed to invade the left masseter muscle and left medial pterygoid muscle, (C) T1 axial MRI revealed mass originate from the angle of mandible and invaded the adjacent muscle medially and laterally.

A facial and neck MRI was conducted to determine the extent of tumor involvement in the bone marrow and to rule out regional metastasis to lymph nodes, particularly the supraclavicular lymph nodes. The MRI revealed a 3.7 x 3.8 cm mass lesion originating from the angle of the mandible on the left side, associated with a soft tissue component involving both sides of the bone. The mass was observed to invade the left masseter muscle and left medial pterygoid muscle (Figure 3). The final diagnosis was FO arising from the left angle of the mandible, with no evidence of regional metastasis to lymph nodes.

The surgical treatment included left-sided segmental mandibulectomy (tumor resection with a 1.5 cm safety margin). Postoperatively, complete excision with adequate safety margins on bone and closed margins on soft tissue was achieved.

**Figure 4:**
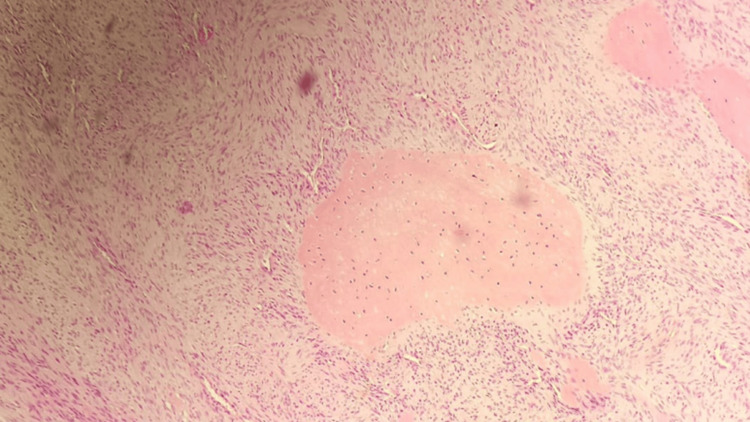
The deep part of the tumor represents rare and focal osteoid formation (low magnification).

**Figure 5:**
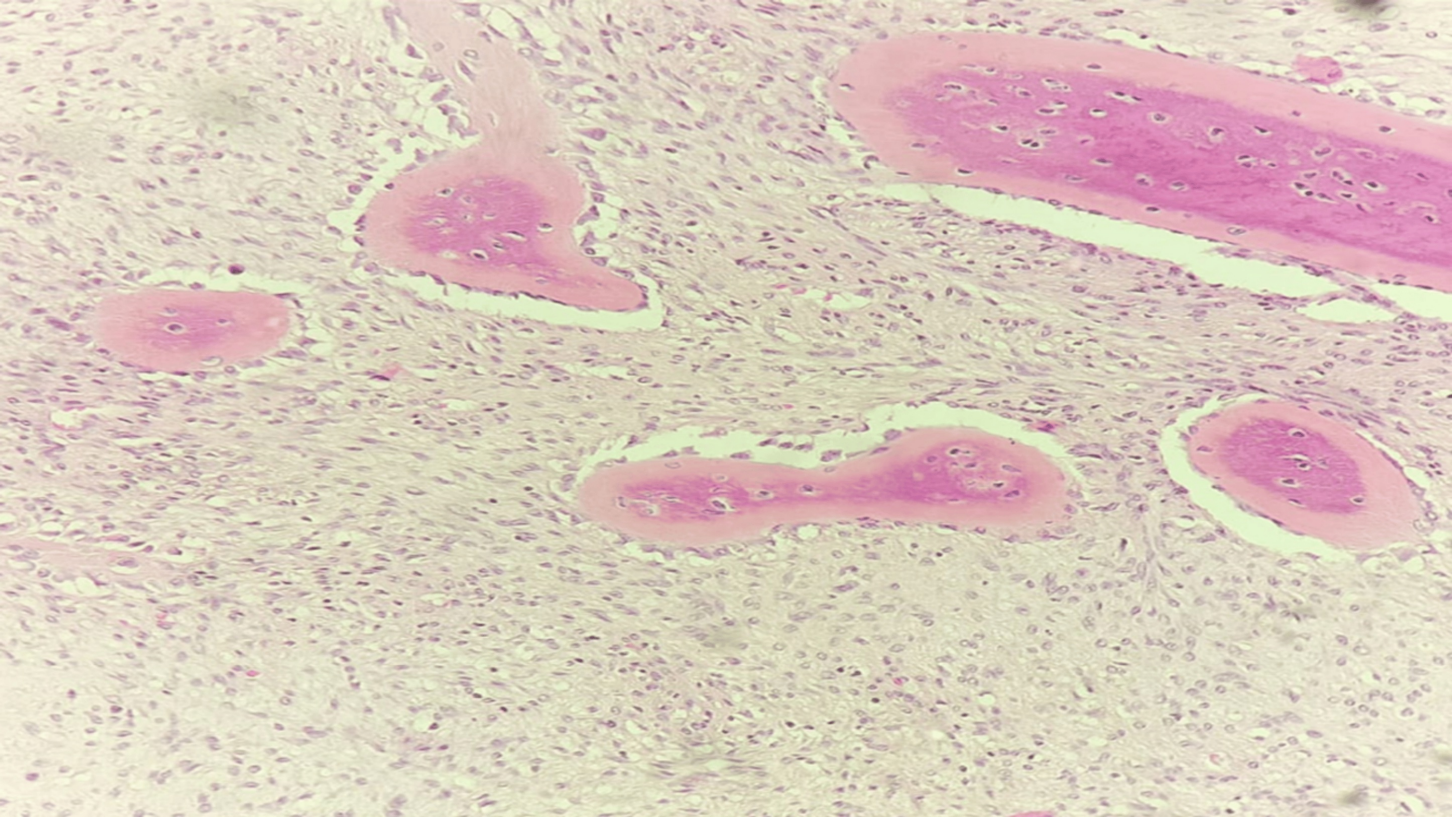
The deep part of the tumor represents rare and focal osteoid formation (high magnification).

The excised specimen was fixed in formalin, paraffin-embedded, and stained with hematoxylin and eosin. Microscopic examination revealed a predominantly spindle cell tumor with moderate nuclear atypia and mitosis, arranged in fascicles and occasional storiform patterns. Osteoid production was minimal and focal (Figures 4, 5). The tumor exhibited moderate vascularity, with some areas resembling a hemangiopericytoma-like pattern and evidence of extravasated red blood cells. Consequently, the diagnosis of FO was established. Following a four-week post-surgery period, the patient was referred to the hematology and oncology clinic for adjuvant chemotherapy.

## Discussion

Jaw fibroblastic osteosarcoma (JFO) is particularly the rarest type of JO. Consequently, the number of published case reports is small compared to the more common histological type of JO. JFO arises from the fibrous stroma and is characterized by the presence of osteoid secreted from spindle-shaped cells.

Clinically, JO most commonly presents as the rapid growth of the lesion, pain, and mobility or displacement of the tooth. Radiologically, it can present as a mixed lytic and sclerotic lesion with irregular margins and area of bone destruction. Furthermore, soft tissue extension and the presence of sunburst or Codman’s triangle patterns on radiographs. Although the clinical and radiological findings of the lesions are suggestive of JO [11], this is not the case in JFO. Furthermore, these findings can be seen in jaw pathologies other than JO such as benign tumors, metastatic lesions, or infections. For these reasons, a biopsy and histopathological examination are mandatory for achieving a definitive diagnosis.

In the presented case, the dilemma of FO diagnosis became obvious during the histopathological examination. The reason was the small-sized, peripheral biopsy specimen, which did not represent fibroblastic changes. Also, the biopsied tissue showed bland spindled fibroblastic cells to stellate cells with a variably cellular tissue culture-like pattern, accompanied by extravasated red blood cells and pale nuclei with prominent nucleoli. These findings were suggestive of diagnosing the lesion as a benign soft tissue spindle-cell lesion called nodular fasciitis. However, because incapability to correlate the CT findings and clinical signs of the presented lesion, the surgeon and histopathologist consensus to perform additional incisional biopsies from different parts of the tumor, particularly from deeper locations. In the second examination, the presence of osteoid formation was the key finding in the second specimen that changed the final diagnosis into JFO.

The diagnostic dilemma of JFO lies in its resemblance to proliferative reactive lesions, like nodular fasciitis since both share some overlapping features [12,13]. However, nodular fasciitis is a benign reactive lesion characterized by rapid growth, pain, and a nodular appearance. It may present histological features resembling malignancy, including cellular atypia and mitotic activity, which can mimic sarcomas such as JFO. However, the immunohistochemistry for both nodular fasciitis and JFO may show positivity for vimentin, smooth muscle actin (SMA), and desmin [14,15]. The JFO may demonstrate osteoid production and positivity for osteocalcin or other osteogenic markers [16,17]. Unlike nodular fasciitis, JFO has a malignant feature such as cellular atypia, hyperchromasia, and increased mitotic activity [18]. Also, the presence of osteoid or bone formation within the tumor matrix is a hallmark feature of JFO diagnosis [19]. Finally, distinguishing between these two entities histopathologically relies on recognizing the presence or absence of malignant features such as bone formation, invasive growth, and cellular atypia.

## Conclusions

In conclusion, the diagnostic dilemma between FO and nodular fasciitis underscores the importance of comprehensive clinical assessment and histopathological evaluation. While both conditions may present with similar clinical features, including rapidly growing soft tissue masses, careful consideration of histopathological features, immunohistochemical staining, radiographic findings, and clinical history is essential for accurate diagnosis and appropriate management. Histopathological examination remains the cornerstone for distinguishing between FO and nodular fasciitis. The FO demonstrates malignant spindle cells with osteoid production, while nodular fasciitis is characterized by myofibroblastic proliferation without osteoid formation. Immunohistochemical staining and radiographic imaging further aid in the differentiation process. Consultation with a multidisciplinary team, including pathologists, radiologists, and oncologists, is paramount to ensure accurate diagnosis and optimal treatment planning tailored to the individual patient. Further research and clinical experience are warranted to refine diagnostic criteria and improve outcomes for patients presenting with this challenging diagnostic dilemma.
